# Rho Signaling Participates in Membrane Fluidity Homeostasis

**DOI:** 10.1371/journal.pone.0045049

**Published:** 2012-10-05

**Authors:** Daniel Lockshon, Carissa Perez Olsen, Christopher L. Brett, Andrei Chertov, Alexey J. Merz, Daniel A. Lorenz, Marc R. Van Gilst, Brian K. Kennedy

**Affiliations:** 1 Department of Biochemistry, University of Washington, Seattle, Washington, United States of America; 2 Buck Institute for Age Research, Novato, California, United States of America; 3 Fred Hutchinson Cancer Research Center, Seattle, Washington, United States of America; 4 Sonoma State University, Rohnert Park, California, United States of America; Institute of Developmental Biology and Cancer Research, France

## Abstract

Preservation of both the integrity and fluidity of biological membranes is a critical cellular homeostatic function. Signaling pathways that govern lipid bilayer fluidity have long been known in bacteria, yet no such pathways have been identified in eukaryotes. Here we identify mutants of the yeast *Saccharomyces cerevisiae* whose growth is differentially influenced by its two principal unsaturated fatty acids, oleic and palmitoleic acid. Strains deficient in the core components of the cell wall integrity (CWI) pathway, a MAP kinase pathway dependent on both Pkc1 (yeast's sole protein kinase C) and Rho1 (the yeast RhoA-like small GTPase), were among those inhibited by palmitoleate yet stimulated by oleate. A single GEF (Tus1) and a single GAP (Sac7) of Rho1 were also identified, neither of which participate in the CWI pathway. In contrast, key components of the CWI pathway, such as Rom2, Bem2 and Rlm1, failed to influence fatty acid sensitivity. The differential influence of palmitoleate and oleate on growth of key mutants correlated with changes in membrane fluidity measured by fluorescence anisotropy of TMA-DPH, a plasma membrane-bound dye. This work provides the first evidence for the existence of a signaling pathway that enables eukaryotic cells to control membrane fluidity, a requirement for division, differentiation and environmental adaptation.

## Introduction

Lipid bilayers must remain impermeable to even the smallest ions, yet must also maintain sufficient disorder to preserve the fluidity required for dynamic processes such as migration of proteins within the membrane. Such homeostasis is critical for proper receptor signaling, membrane curvature, endocytosis, exocytosis, and organelle biogenesis. In several bacterial species the molecular mechanisms that control membrane fluidity have been described in detail [1]. For example, the increase in width of the *B. subtilis* cell membrane that accompanies loss of fluidity induces autophosphorylation of DesK, a histidine kinase sensor [2], and the ensuing phosphorylation of the transcriptional activator DesR elicits transcription of *des*, the sole acyl desaturase. The resulting increase in monounsaturated relative to saturated fatty acids within *B. subtilis* phospholipid disrupts acyl chain packing to restore fluidity. In eukaryotes, while the compensatory changes in phospholipid acyl composition that occur in response to alterations in temperature (often termed homeoviscous adaptation [3]) are well established [4], [5], [6], the signaling pathways that achieve such homeostasis have not been identified.

**Figure 1:**
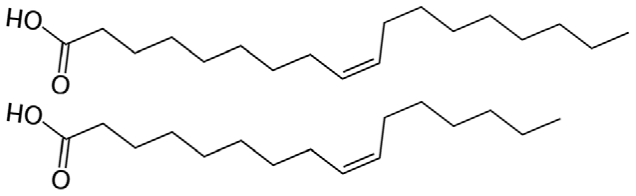
Structures of C18:1 (oleic acid; C18:1Δ9) and C16:1 (palmitoleic acid; C16:1Δ9).

*Saccharomyces cerevisiae* is an ideal system for investigating the signaling that enables eukaryotic membrane fluidity homeostasis. Its genetic utility is complemented by the relative simplicity of its phospholipid fatty acid content [7], an important determinant of membrane fluidity [8]. Our previous work identified ∼130 genes needed for optimal growth in the presence of oleic acid (C18:1Δ9). Surprisingly, two C18:1-sensitive (C18:1^S^) mutants were unaffected by palmitoleate (C16:1Δ9), the other major unsaturated fatty acid in yeast phospholipid [9]. Such divergent effects of two monounsaturated fatty acids that differ minimally in chain length (Figure 1) led us to speculate that these two mutants are impaired in their ability to regulate membrane fluidity.

By comprehensively identifying deletion strains whose growth is differentially influenced by the two fatty acids, we have now identified ∼100 mutants whose growth is sensitive to one fatty acid yet unaffected by, or in some cases even stimulated by, the other. Of the six signaling protein mutants exhibiting this phenotype, all but one lack core components of the cell wall integrity (CWI) pathway, a well-characterized yeast MAP kinase pathway [10]. Additional experiments confirmed the involvement of Pkc1 and other CWI pathway components, yet also demonstrated that upstream regulators and a downstream effector of the CWI pathway do not play a role in this differential fatty acid growth phenotype, showing that this membrane fluidity homeostasis pathway and the CWI pathway are overlapping yet distinct. Measurement of mutants' phospholipid acyl group composition demonstrated that this pathway influences membrane composition. Fluorescence anisotropy measurements directly showed that activation of the pathway decreased fluidity of the plasma membrane (PM). Thus, while yeast cell wall integrity is monitored by the CWI pathway, a distinct yet overlapping membrane fluidity homeostasis pathway preserves the integrity of the adjacent PM and perhaps that of additional yeast membranes.

## Results

### Mutants with fatty acid chain length-dependent growth phenotypes

In a previous study, we screened a set of 4773 haploid strains, each missing a non-essential gene, for growth inhibition by C18:1. Two such C18:1^S^ strains (*ilm1Δ* and *sap190Δ*) were unaffected by C16:1 [9]. This result prompted a second screen, described here, to compare the effects of these two most abundant yeast fatty acids on growth of the entire collection of single gene knockout strains (the two other abundant yeast fatty acids, C16:0 and C18:0 were not examined because their saturated alkyl chains make them poorly soluble). By comparing growth on plates containing C18:1, C16:1, or neither (Figure S1), we identified 53 additional mutants whose growth was inhibited by C18:1 but not by C16:1. Conversely, 54 strains were growth-inhibited by C16:1 but not by C18:1. Subset of these strains, classified into functional groups, are shown in Table 1 (Table S1 provides a complete list).

**Table 1:** Thirty two of the deletions identified by the screen.

grouping	strain	function	C16:1Δ9	C18:1Δ9
signaling	*bck1Δ*	MAP3K	↓↓↓	↑↑
	*sac7Δ*	GAP of Rho1		↓↓↓
	*slt2Δ*	MAP kinase	↓↓↓	
	*ste11Δ*	MAP3K	↓↓	↓↓
	*swi6Δ*	cell cycle	↓↓↓	↓
	*tus1Δ*	GEF of Rho1	↓↓↓	↑↑↑
cytoskeleton	*app1Δ*	actin patch	↓↓↓	
	*arp1Δ*	dynactin	↓↓↓	↓
	*bem1Δ*	polarity	↓↓↓	↓
	*cnm67Δ*	SPB	↓↓	↑↑↑
	*dyn2Δ*	dynein	↓↓↓	
	*dyn3Δ*	dynein	↓↓	
	*jnm1Δ*	dynactin	↓↓↓	
	*num1Δ*	dynein-MT	↓↓↓	
	*vrp1Δ*	actin patch	↓↓↓	↑↑
lipid metabolism	*eci1Δ*	β-oxidation	↓↓↓	
	*erg6Δ*	erg. biosyn.	↓↓↓	↓
	*fox2Δ*	β-oxidation	↓↓↓	
	*pot1Δ*	β-oxidation	↓↓↓	↓
	*pox1Δ*	β-oxidation	↓↓↓	
membrane	*gup1Δ*	GPI	↓↓↓	↓
	*rvs161Δ*	curvature	↓↓↓	
	*vam7Δ*	SNARE	↓↓↓	↓
mitochondrion	*aim11Δ*	inheritance	↓↓↓	↑↑↑
	*atp11Δ*	ATP syn.	↓↓↓	
	*atp18Δ*	F1-F0	↑↑↑	
	*mdm31Δ*	inheritance	↑↑↑	↓↓↓
	*mrpl10Δ*	ribosome	↓↓↓	
	*mrs2Δ*	ion transport	↑↑	↓↓↓
	*nfu1Δ*	Fe/S	↓↓↓	
	*phb2Δ*	chaperone	↓↓↓	
	*por1Δ*	ion channel	↑↑	

4773 isogenic MATα deletion strains, each deleted in a single non-essential gene [62], were applied robotically to semi-synthetic medium containing either 0.1% C16:1, 0.1% C18:1, or neither, and grown 2 weeks at 25° [9]. Of the total of 212 strains identified as inhibited (↓) or stimulated (↑) in growth to varying degrees (the greater the effect, the more arrows) by C16:1 or C18:1, growth of 130 showed substantial differential effects of C16:1 and C18:1 on growth (47 were C16:1-inhibited and unaffected by C18:1, 7 were C16:1-inhibited and C18:1-stimulated, 52 were C18:1-inhibited and unaffected by C16:1, 3 were C18:1-inhibited and C16:1-stimulated, 19 were stimulated in growth by C16:1 yet unaffected by C18:1, and 2 were stimulated in growth by C18:1 yet unaffected by C16:1). The growth of 71 of the remaining 82 was inhibited by both C16:1 and C18:1 (37 of these were equally sensitive to both) and the final 11 were stimulated in growth by both fatty acids. See Table S1 for the complete data set.

**Figure 2:**
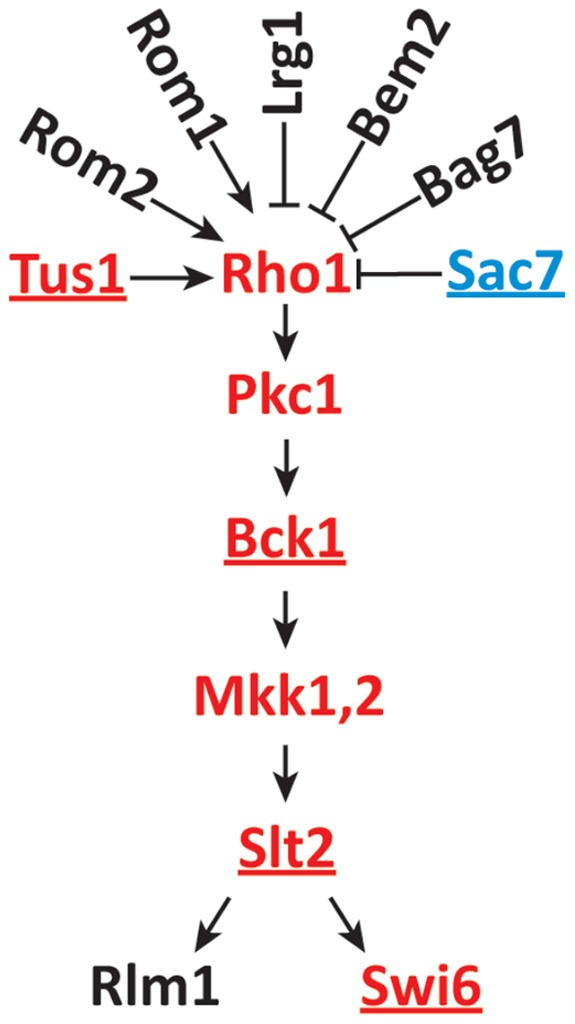
Rho1 signaling in the context of the CWI pathway. All proteins shown have been implicated in the CWI pathway or in control of Rho1. All three known Rho1 GEFs (Rom1, Rom2, and Tus1) and all four known Rho1 GAPs (Lrg1, Bem2, Bag7, and Sac7) are shown. Underlining indicates proteins found in the screen. Proteins drawn in red are those which when mutated cause sensitivity to C16:1 but not C18:1. Sac7 is drawn in blue because *sac7Δ* instead causes C16:1^R^ C18:1^S^.

Most notable among the identified mutants were ten whose growth was affected in opposite directions by C16:1 *vs.* C18:1. Growth of three C18:1^S^ strains was stimulated by C16:1, and growth of seven C16:1^S^ strains was stimulated by C18:1. Among the latter are *bck1Δ* and *tus1Δ*. Bck1 is the MAP3K (MAP kinase kinase kinase) component of the cell wall integrity (CWI) signaling pathway, one of four standard MAP kinase pathways in *Saccharomyces* [11]. This pathway, shown in Figure 2, senses yeast cell wall damage, activating a transcriptional cell wall repair response [12], [13]. Tus1 [14] is a guanyl nucleotide exchange factor (GEF) that activates Rho1, one of six Rho-GTPases in *S. cerevisiae*. Of the six, Rho1 is most similar in sequence and function to RhoA, the well-studied mammalian regulator of actin cytoskeleton dynamics [15]. Among other functions, yeast Rho1 activates Pkc1, yeast's sole protein kinase C [13], [16], [17]. Activation of Pkc1 enables it to phosphorylate and thereby activate Bck1 in the CWI pathway [18].

Three additional CWI pathway components, Slt2, Swi6, and Sac7, were also shown in the screen to prevent differential effects of C16:1 and C18:1 on growth. Deletion of either *SLT2* or *SWI6* conferred C16:1- but not C18:1-growth sensitivity. Slt2, a MAP kinase, functions in the CWI pathway to directly phosphorylate both Rlm1 and Swi6 [19], [20]. Rlm1 is a transcription factor that, in its phosphorylated form, stimulates expression of cell wall metabolism and repair genes [21], [22]. Swi6 is one of two subunits of SBF, a transcription factor involved in both the CWI pathway and cell cycle control [20], [23]. Active, phosphorylated Slt2 interacts with SBF first directing it into the nucleus (by non-catalytically interacting with Swi4, the other SBF subunit) and subsequently by phosphorylating Swi6 causing SBF to re-enter the cytoplasm [20], [24], [25]. Third, *sac7Δ* in our screen exhibited C18:1^S^ growth yet was unaffected by C16:1, a phenotype opposite that of the four mutants discussed above. Sac7, assigned to the CWI pathway based on the ability of *SAC7* deletion to cause Slt2 activation/phosphorylation [26], is one of four GTPase activation proteins (GAPs) for Rho1 [27]. Intriguingly, *SAC7* was first identified by a point mutation (and subsequently as a deletion) which suppressed *act1-4*, a ts allele of the sole yeast actin gene [28]. On that basis, *sac7Δ* could also be put in the “cytoskeleton” category in Table 1. We hypothesized that the opposite phenotypes of *sac7Δ* and *tus1Δ* in our screen were a manifestation of the opposite influence of Sac7 and Tus1 on Rho1. These initial results thus prompted us to examine additional components of the CWI.

### Additional signaling genes contribute to fatty acid growth phenotypes

**Figure 3:**
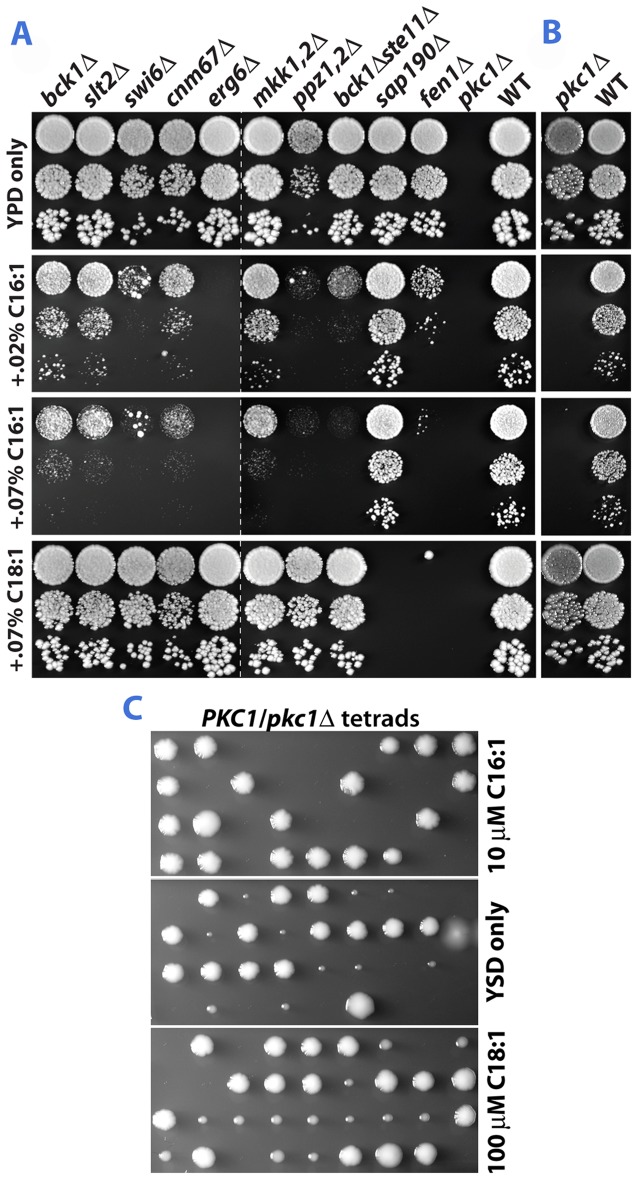
C16:1 and C18:1 differentially affect the growth of mutants. (A) Three, ten-fold dilutions of yeast suspensions were plated on YPD+1% tergitol containing the indicated levels of free fatty acids. The five left-most strains were identified by the screen. The sixth, seventh, and eighth strains from the left were constructed by mating strains with single deletions, sporulating the diploids and dissecting tetrads. *sap190Δ* (C16:1^R^ C18:1^S^) and *fen1Δ* (C16:1^S^ C18:1^S^), both identified by the screen, are included as controls. Growth was at 30° for 3 days. (B) YPD+1% tergitol containing 1 M sorbitol. (C) *pkc1Δ* strains are acutely sensitive to C16:1. Asci from a sporulated *PKC1*/*pkc1Δ* diploid were dissected on 3 slabs of YPD medium containing 1 M sorbitol (YSD; 3 ml per microscope slide) and germinated at 30° for 20 hr. to give colonies containing between 10 and 200 cells. Agar slabs were then slid onto YSD plates containing C16:1 or C18:1 and grown at 30° for 4 days. This two-step procedure allowed attribution of colony size to the effect of fatty acids on vegetative growth rather than to an effect on spore germination. Spore viability on the 3 slabs ranged from 75 to 80%.

Figure 3 shows the effects of C16:1 and C18:1 on growth of several strains identified in the screen. Also shown is the growth of deletion strains not found in the screen for a variety of reasons, yet predicted to also show differential fatty acid sensitivity. Pkc1 could not be identified in our screen since it is required for viability in the low osmolarity medium [13] used to maintain the deletion collection (Figure 3A and 3B). Sporulation of a *PKC1/pkc1* heterozygous diploid and dissection of tetrads on high osmolarity medium enabled us to demonstrate that *PKC1* deletion caused acute C16:1^S^ (Figure 3C), an effect that preceded the accumulation of suppressors of slow growth (Figure S2). In contrast, as was also seen for *tus1Δ* and *bck1Δ*, growth of the colonies derived from the *pkc1Δ* spores was stimulated by C18:1.

At least two steps in the CWI pathway are catalyzed by functionally redundant proteins: the MAP kinase kinase step (Mkk1 and Mkk2) [29], and two phosphatases whose position in the pathway has not yet been established (Ppz1 and Ppz2) [30]. We therefore constructed double mutants to test the role of these components on fatty acid sensitivity. Deletion of both *MKK1* and *MKK2* caused C16:1- but not C18:1-sensitivity (Figure 3A). The presence of both Ppz1 and Ppz2, partially redundant phosphatases, was also required for optimal growth in the presence of C16:1 but not C18:1. Ste11 is a second MAP3K, in addition to Bck1, found by our screen. Upon retesting, growth of *ste11Δ* was inhibited by C16:1 but not by C18:1 (Figure S3). Ste11 is the sole MAP3K of three other yeast MAP kinase pathways (mating pheromone response, pseudohyphal growth response, and high osmolarity/glycerol response), and possibly also plays a subordinate role in the CWI pathway [31]. Deletion of *STE11* potentiates the C16:1-specific growth inhibition due to *BCK1* deletion alone (Figure 3A). Ssk22 is a third yeast MAP3K, assigned solely to the high osmolarity/glycerol response pathway. *bck1Δ ste11Δ ssk22Δ* is even more sensitive to C16:1 than is *bck1Δ ste11Δ* (Figure S3).

The following signaling proteins were not detected in our screen: Rlm1, the transcription factor for cell wall repair genes [21]; Swi4, the other component of SBF; Rom1 and Rom2 [32], the only other GEFs (in addition to Tus1) known to activate Rho1 (*rom1,2Δ* is inviable); the Rho1 GAPs Bem2 [33], [34], [35], Bag7 [27] and Lrg1 [36], [37], [38]; Rho2, with which Rho1 shares some functions [27]. Retesting the MATα strains from the collection confirmed this negative data (not shown). The inability of either fatty acid to influence the growth of these eight deletion strains strongly suggests that cell wall damage and fatty acid-induced stress participate in distinct signaling mechanisms that converge at Rho1.

### Membrane fluidity homeostasis is impaired in mutants differentially sensitive to C18:1 and C16:1

In addition to the extent of their desaturation and branching, the length of phospholipid acyl chains is an important determinant of membrane fluidity. Shortening of acyl chains decreases the hydrophobic forces that keep the bilayer intact thereby increasing fluidity. *S. cerevisiae* is known to adjust chain length in response to changes in growth temperature: Suutari *et al.* [39] reported a 1.7-fold decrease in C16 to C18 ratio when growth temperature was raised from 10° to 35° while Martin *et al.* [40] showed a 2.1-fold decrease when elevating growth temperature from 15° to 34° (Notably, both studies found temperature to have no effect on the ratio of saturated to unsaturated phospholipid acyl groups.) The increase in relative amount of C18 with increasing growth temperature enables the cell to counter the concomitant increase in fluidity. Given that fatty acids supplied to *S. cerevisiae* growth medium are readily incorporated into phospholipid [41], we hypothesized that effects on membrane fluidity might explain the differential effects of C16:1 and C18:1 on growth.

**Figure 4:**
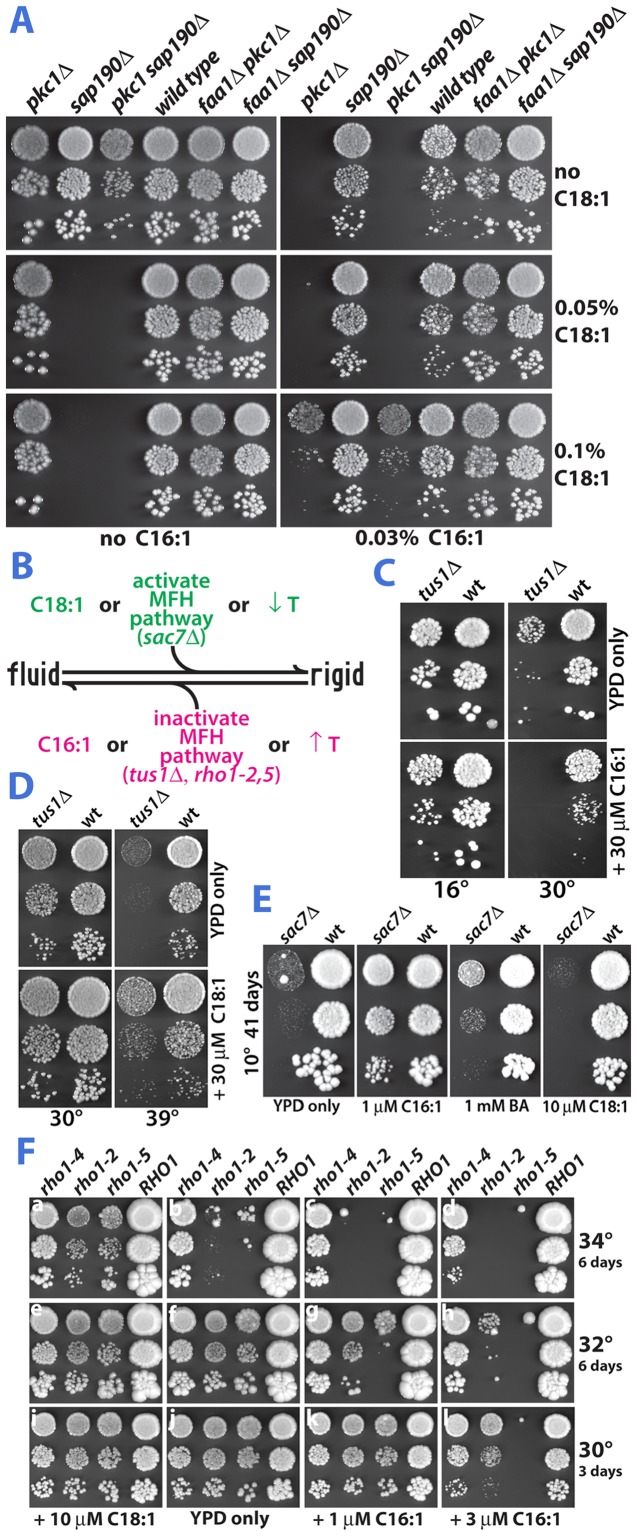
Growth properties of mutants are consistent with impaired regulation of membrane fluidity. (A) The effects of C16:1 and C18:1 on growth counteract each other. Six strains were grown on YPD medium containing 1 M sorbitol and 1% tergitol supplemented with C16:1 (right panels) or not (left panels) and/or two levels of C18:1 (two bottom pairs of panels) or not (upper panels) and grown at 30° for 3 days. (B) Representation of the interplay between fatty acid composition, the proposed membrane fluidity homeostasis (MFH) signaling pathway, and temperature (T) on membrane fluidity. (C) C16:1-sensitivity of *tus1Δ* is suppressed by growth at 16°. Yeast were grown on YPD with or without C16:1 for 11 days (16°) or 2 days (30°). (D) The ts of *tus1Δ* is suppressed by C18:1. Growth on YPD with or without C18:1 was for 3 days. (E) The cs of *sac7Δ* is suppressed by either C16:1 or BA. YPD medium was supplemented with C16:1 or C18:1 or with benzyl alcohol (BA). (F) Growth of only one class of *rho1^ts^* strains is inhibited by C16:1 at permissive temperatures, and is enhanced by C18:1 at a semi-permissive temperature.

This model predicts that supplementation of media with C18:1 counteracts the effect of C16:1 on growth. Indeed, C18:1 suppressed growth inhibition of *pkc1Δ* by C16:1 (Figure 4A, top *vs.* bottom panels). Conversely, C16:1 suppressed the C18:1 growth inhibition of *sap190Δ* (left *vs.* right, top *vs.* middle and bottom panels), chosen because it exhibits C18:1- but not C16:1-sensitivity [9]. As would be expected, either fatty acid alone inhibited growth of the *pkc1Δ sap190Δ* double knockout. Strikingly however, simultaneous addition of both C16:1 and C18:1 restored its growth. This third result rules out competition for uptake as the basis of the antagonistic effects of the two fatty acids on toxicity. Incorporation of fed fatty acids into phospholipid requires Faa1, the main (palmit)oleyl-CoA synthetase in yeast [42]. Deletion of *FAA1* suppressed all effects of fatty acids on growth (Figure 4A), indicating that C16:1 and C18:1 influence growth of the mutants by their incorporation into phospholipid (not merely by non-covalent binding to the plasma membrane, for example).

Membrane fluidity is enhanced at high temperature. Thus, as diagrammed in Figure 4B, the hypothesis explains the interplay between the effects of growth temperature, fatty acids, and deletion of *TUS1* (Figure 4C and 4D) as follows: First, lowering growth temperature from 30° to 16° curtails the ability of C16:1 (a fluidizer) to inhibit growth of *tus1Δ* (Figure 4C), the result expected for a strain with hyper-fluidized membrane. Second, an increase in growth temperature from 30° to 39° enhances the ability of C18:1 (a rigidifier relative to C16:1) to stimulate the growth of *tus1Δ* (Figure 4D), thereby recapitulating at 39° the C18:1-stimulation of *tus1Δ* growth seen in the screen. Third, Figure 4C and 4D also confirm [14] the temperature sensitivity (ts) of *tus1Δ*, a property that is now partially explained by excessive membrane fluidity.

Likewise, fluidity decreases at lower temperature. The study that first described Sac7, the Rho1 GAP whose ablation in our screen caused C18:1- but not C16:1-sensitivity, reported *sac7Δ* to be cold-sensitive (cs) [28]. This cs phenotype, as well as the opposing roles of Sac7 *vs.* Tus1 on Rho1 GTPase activity, led us to suspect that this mutation causes excessive rigidification of membrane. Previously, membrane rigidification was proposed to account for the cs of *Listeria monocytogenes* mutants which are defective in the synthesis of branched fatty acids [43]. Figure 4E shows that 10° growth of *sac7Δ* was enhanced by merely 1 µM C16:1 (at 30° *SAC7* has no influence on growth). Benzyl alcohol (BA), a well-established membrane fluidizer [44], [45], also suppressed the cs of *sac7Δ*. Conversely, C18:1 inhibited growth of *sac7Δ*, both in the screen and at 10°. Two additional effects of BA on growth (Figure S4) further support our model: Inhibition of *pkc1Δ* growth by BA was suppressed by C18:1. Second, BA reversed the C18:1 inhibition of growth of *sap190Δ*.

Point mutations in *RHO1* itself, an essential gene, also caused differential effects of C16:1 *vs.* C18:1 on growth (Figure 4F). Six *RHO1* ts alleles, isolated and characterized by Saka *et al.* [46], fall into two classes. At the restrictive temperature (37°), only class-A alleles (*rho1-2* and *rho1-5*) are deficient in the phosphorylation of Slt2 *in vivo* whereas only class-B alleles (*rho1-4* and three others not used here) were deficient in the synthesis of 1,3-β-glucan (a yeast cell wall component), a second essential regulatory Rho1 function. When grown at 30°, a permissive temperature for these three ts alleles, 3 µM C16:1 completely inhibited growth of *rho1-5* and partially inhibited growth of a strain bearing *rho1-2*, the weaker class-A allele [46] (Figure 4F, j *vs.* l). At slightly higher growth temperature (32°), merely 1 µM C16:1 inhibited growth of *rho1-5* (f *vs.* g) while growth of *rho1-2* was now inhibitable by 3 µM C16:1 (f *vs.* h). Conversely, lower growth temperature (23°) caused C16:1 to be less effective in inhibiting both of these class-A strains (Figure S5). The influence of temperature on growth inhibition of these two class-A *rho1* strains by C16:1 is analogous to the result using *tus1Δ* (Figure 4B). In contrast, growth of the class-B mutant (*rho1-4*) was not inhibited by C16:1, even at higher (34°) temperature (b, c and d). As with the deletion strains that were surmised to be defective in membrane fluidity signaling, C18:1, the membrane rigidifier, had an opposite effect on growth of the two class-A *RHO1* ts strains. At 34°, a semi-permissive growth temperature for *rho1-2* and *rho1-5*, 10 µM C18:1 enhanced growth (Figure 4F, a *vs.* b). As expected however, C18:1 is incapable of enabling growth of the *rho1-4* strain at 36°, its restrictive growth temperature (Figure S5).

Taken together, the results in Figure 4 are interpreted as follows: blockage of Rho1 signaling impairs the cell's ability to resist the lethal degree of membrane fluidization that results from either temperature increase or excessive incorporation of C16:1 into phospholipid. Conversely, increasing Rho1 activity by deleting *SAC7* prevents the cell from relieving the excessive membrane rigidification that results from lower temperature or from excessive C18:1 phospholipid content. The data in Figure 4F directly implicates Rho1 itself in membrane fluidity homeostasis and confirms the lack of an effect of *rho2Δ*, described above. Thus, growth of a variety of mutants under a range of conditions suggests that Rho1, its GEF Tus1, and its GAP Sac7 participate in the control of membrane fluidity.

### Membrane properties of mutants with fatty acid growth phenotypes

**Figure 5:**
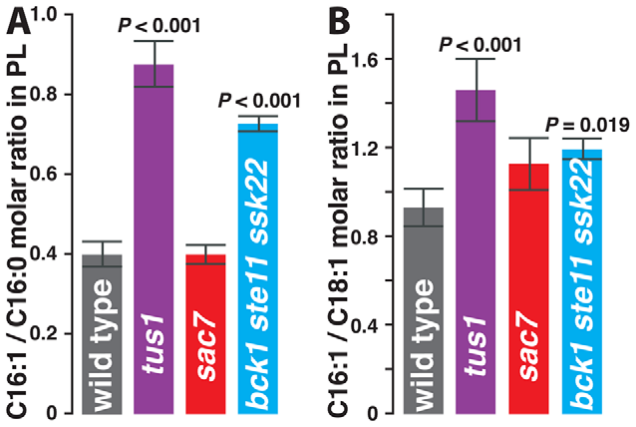
Acyl chain content of PL is influenced by signaling proteins. Total PL from each of four strains was purified in multiple experiments and acyl chain content quantified (Table S2 contains the complete data set). Mutations caused statistically significant changes in the molar ratios of (A) C16:1/C16:0 and of (B) C16:1/C18:1, but in neither the C18:1/C18:0 nor the C16:0/C18:0 ratios (Figure S5). Error bars are the standard error of the mean. P values compared to wild type by paired, two-tailed Student's t-test are shown for *tus1Δ* and for *bck1Δ ste11Δ ssk22Δ*.

The four predominant fatty acids in yeast phospholipid are C16:1, C18:1, C16:0, and C18:0. We directly examined the influence of mutations on these species by quantifying fatty acid methyl esters derived from total yeast phospholipid extracted from logarithmically-grown cultures. Deletion of *TUS1* caused substantial increases in both the C16:1 to C16:0 ratio (>2-fold) and the C16:1 to C18:1 ratio (>1.5-fold, Figure 5). The C18:1/C18:0 and C16:0/C18:0 ratios were perhaps also slightly increased in *tus1Δ* (Figure S6). Phospholipid of *bck1Δ ste11Δ ssk22Δ*, which also showed substantial C16:1^S^ growth (Figure S3), was also analyzed. Similar to *tus1Δ*, this triple MAP3K knockout also showed significant increases in C16:1/C16:0 and C16:1/C18:1 ratios relative to wild type (Figure 5). These increases in both C16 desaturation and in the levels of shorter *vs.* longer unsaturated fatty acids are consistent with an increase in fluidity in *tus1Δ* and *bck1Δ ste11Δ ssk22Δ*. Interestingly, no significant changes in these two ratios were found for *sac7Δ*, although small but significant decreases in the other two ratios (C18:1/C18:0 and C16:0/C18:0), relative to wild type, were observed (Figure S6).

**Figure 6:**
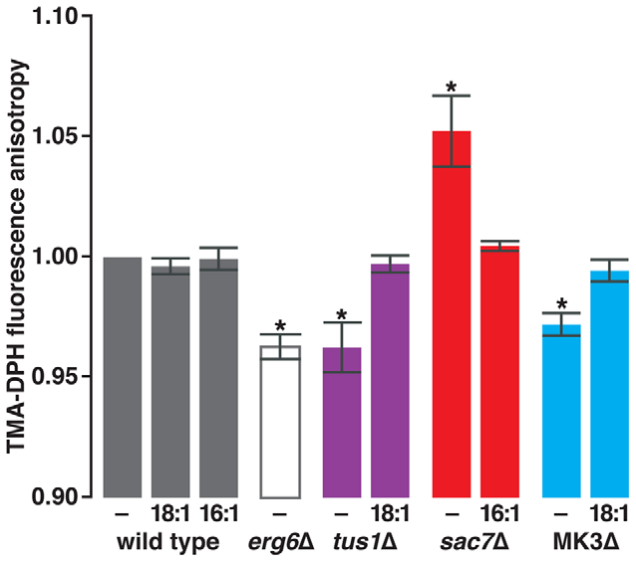
Losses of Rho1 GEF and GAP activities have opposing effects on PM fluidity. Using TMA-DPH as a probe, fluorescence anisotropy was performed on wild type, *erg6Δ*, *tus1Δ*, *sac7Δ*, and *bck1Δ ste11Δ ssk22Δ* (“MK3Δ”), grown logarithmically in the absence (−) or presence of either 10 µM C18:1 or 10 µM C16:1. Addition of up to 100-fold more C18:1 or C16:1 had no additional effect. Anisotropy values are expressed relative to wild type (in the absence of either soap) and are shown as the mean ± S.E.M calculated from at least 3 independent experiments. *, *p*≤0.005 *vs.* wild type by paired, two-tailed Student's t-test.

Membrane fluidity *in vivo* can be assayed by fluorescence anisotropy, the degree to which the fluorescence of a membrane-embedded probe, excited by polarized light, is depolarized due to its motion within the membrane. The trimethylammonium group of the fluorescent probe we employed (trimethylammonium diphenylhexatriene, TMA-DPH) prevents its entry into the cell thereby permitting measurement of fluidity exclusively of the PM [47]. As was first observed by Sharma [48], we confirmed that deletion of *ERG6* causes an increase in TMA-DPH depolarization *i.e.*, a decrease in fluorescence anisotropy (Figure 6), interpreted as an increase in PM fluidity. Erg6 is a methyltransferase that rearranges side chains in a late step in the biosynthesis of ergosterol [49], the predominant yeast sterol [50]. The decrease in PM fluidity of *erg6Δ* supports a large body of evidence demonstrating the influence of sterols and their side chains on fluidity *in vitro* (reviewed in [51]). Since deletion of *ERG6* also caused C16:1^S^, C18:1-insensitive growth (Figure 3A), the observed increase in of *erg6Δ* fluidity lends support to our hypothesis that hyper-fluidity underlies C16:1-specific growth sensitivity.

Figure 6 also portrays the measurements of anisotropy of PM-bound TMA-DPH fluorescence in cells deficient in Rho1 signaling. Deletion of *TUS1* caused a substantial decrease in anisotropy, whereas deletion of *SAC7* caused anisotropy to substantially increase. Tus1 (a GEF) and Sac7 (a GAP) are known to positively and negatively influence Rho1 activity, respectively. Hence, inactivation of Rho1 by deletion of *TUS1* increases PM fluidity (a conclusion also reached from the growth data in Figure 4C and 4D) and activation of Rho1 by deletion of *SAC7* decreases PM fluidity (as inferred by Figure 4E). The substantial effect of Sac7 on fluidity contrasts with its insignificant effect on the acyl group ratios shown in Figure 5. The dependence of fluidity on an additional cellular property would resolve this contradiction. The involvement of Sac7 in some way with the actin cytoskeleton, as well as the differential effect of C16:1 and C18:1 on *vrp1Δ* (Table 1), *las17Δ*, and *sac6Δ* (unpublished data) suggests that the actin cytoskeleton may also participate in modulating fluidity.

Measuring TMA-DPH fluorescence depolarization in yeast grown in the presence of C16:1 or C18:1 further established the relationship between mutations, fatty acids, and fluidity. The inhibition and enhancement of growth of *tus1Δ* by C16:1 and C18:1, respectively, was interpreted as evidence of the excessive fluidity of their membranes (Figure 4C and 4D). Indeed, Figure 6 shows that growth of *tus1Δ* in the presence of growth-stimulatory C18:1 (10 µM) caused a decrease in PM fluidity to the level seen in wild type cells. Similarly, the excessive PM fluidity of *bck1Δ ste11Δ ssk22Δ* (MK3Δ), a second C16:1^S^ strain, decreased to wild type levels when grown in the presence of C18:1. Conversely, growth of *sac7Δ* in the presence of C16:1 caused PM fluidity to increase to wild type levels, thus providing a physical basis for the positive effect of this fatty acid on growth of this strain. Notably, anisotropy in the wild type strain was unchanged by supplementation of the culture with either fatty acid. Thus, when membrane fluidity homeostasis signaling is intact, neither C16:1 nor C18:1 are capable of perturbing PM fluidity. The fluorescence anisotropy data, by its relatively direct measurement of plasma membrane fluidity, demonstrates that the differential influence of C16:1 and C18:1 on growth reflects defects in membrane fluidity control. Thus, our screen has implicated a MAPK pathway and upstream components in membrane fluidity homeostasis.

## Discussion

This study has used genetic methods to identify a set of yeast signaling proteins that prevent C16:1 and C18:1, the main unsaturated fatty acids in yeast phospholipid, from differentially influencing growth. All but one of the signaling proteins identified in the screen are components of the CWI pathway, the yeast Rho1-dependent MAP kinase cascade previously thought to be responsible exclusively for maintaining the integrity of the cell wall. Direct examination of the membrane properties of three strains (*tus1Δ*, *sac7Δ*, and *bck1Δ ste11Δ ssk22Δ*) demonstrated changes in both PL acyl content and in fluorescence anisotropy consistent with altered fluidity. The dependence of mutants' growth properties on temperature, as well as the counteractive effects of C16:1 and C18:1 on growth, support these data. Therefore, at least for these three strains, and probably also for others identified here (Tables 1 and S1), mutations that cause growth to be differentially influenced by C16:1 *vs.* C18:1 interfere with fluidity control of at least one of the essential yeast membrane systems. Thus, the differential sensitivity of yeast growth to C16:1 *vs.* C18:1 provides a method to genetically explore yeast membrane fluidity homeostasis signaling.

Using this growth assay, we have identified what appears to be the first eukaryotic membrane fluidity homeostasis signaling pathway, comprised of the core components of the yeast Rho1/Pkc1/MAPK pathway. While additional work is required to prove that these components constitute an intact fluidity signalling pathway, their known coordination in an additional pathway (CWI) makes this likely. Future work is also necessary to establish whether additional yeast signaling pathways operate to maintain fluidity homeostasis, perhaps of different membrane systems. Nevertheless, the present work has taken the first steps to establish how a eukaryote transmits the signal of excessive membrane fluidity to other parts of the cellular machinery that must act to reverse this potentially lethal state.

Three types of earlier observations support the participation of proteins identified here in fluidity homeostasis: (1) Slt2 MAP kinase activity is stimulated by treatment of cultures with chlorpromazine, by heat shock, and by hypo-osmotic shock [52], all of which promote stretching of the PM. (2) Choline sensitivity and inositol auxotrophy, indicative of defects in membrane biosynthesis, are caused by deletion of *PKC1*, *BCK1*, and *SLT2* [53]. (3) The PM of the following three ts strains ruptures (as judged by the leakage of alkaline phosphatase from cells) when cells are incubated, but not grown (*i.e.*, in the absence of cell wall biosynthesis), at 37°: *rho1-104* [54], *pkc1^P1102S^* (*stt1-1*) [17], and a strain in which *RHO1* is replaced by human RhoA [55]. In addition, support for a role of Pkc1, the sole yeast PKC, in controlling membrane fluidity is also provided by tissue culture knockdown experiments that have recently implicated several mammalian PKC isoforms in fluidity homeostasis [56].

Core components of this sole yeast Rho1/Pkc1/MAPK cascade thus appear to participate in two signaling pathways, one which monitors the integrity of the cell wall and the other which enables membranes to maintain proper fluidity. The two pathways are distinguished by their peripheral components: Rlm1, the well-established transcription factor of the CWI pathway, plays no apparent role in fluidity signaling; transcriptional readouts unique to the membrane fluidity homeostasis pathway are not yet known. Upstream, only a single Rho1 GAP (Sac7) appears to be involved in transmitting fluidity information to the signaling pathway. There is no basis to invoke a role for Sac7 in the CWI pathway since there is little if any genetic or biochemical data linking it to the wall. Thus, based on the data presented here, the GAP function of Sac7 appears to participate exclusively in fluidity control signaling. The sole Rho1 GAP of the CWI pathway, in turn, appears to be Bem2 [34].

Likewise, our data indicates that only a single Rho1 GEF, Tus1, is involved in fluidity sensing. Rom2, as well as Rom1 in a more subsidiary role [57], the only other two known Rho1 GEFs, both function in the CWI pathway. Previously, Tus1 as well was thought to participate in the CWI pathway since high osmolarity (1 M sorbitol) suppressed the ts of *tus1Δ* [14], a phenotype previously observed with *bck1Δ* [12] and also observed by us (data not shown). Schmelzle *et al.* [14] argued that high temperature weakens the cell wall and high external osmolarity prevents the cell from bursting by reducing the osmotic pressure of the PM on the wall. However, we have shown that *ste11Δ* also causes a pronounced osmo-remedial ts phenotype (Figure S7). Thus, since Ste11 at most plays a minor role in CWI signaling [31], osmo-remedial temperature sensitivity is an insufficient criterion for involvement in the CWI pathway. The osmo-remediality of the ts of *bck1Δ* and *tus1Δ* (as well as of *ste11Δ*) is instead more easily explained by the cell-desiccating effect of high external osmolarity. The resulting decrease in PM surface area, according to this explanation, prevents the PM breeching that would otherwise result from the higher PM fluidity in mutants unable to respond to higher temperature by rigidifying their membrane. A recent report from Krause *et al.* [57] supports the participation of Tus1 and Rom2 in distinct pathways. They compared the effects of both deletion and overexpression of *TUS1 vs. ROM2* in two ways: First, using a β-galactosidase reporter, they showed that Rlm1-dependent transcription *increased* upon deletion of *TUS1* and *decreased* upon its overexpression, the opposite result found when *ROM2* was deleted and overexpressed. Second, they reported little overlap between the sets of yeast deletions that cause synthetic sick (or lethal) growth when *tus1Δ vs. rom2Δ* is used to query the set of deletion strains. These data provide strong evidence that the biological functions of Tus1 and Rom2 differ substantially.

A final line of evidence indicating that the CWI and the membrane fluidity homeostasis pathways are distinct is provided by comparing the relative growth-sensitivity of *rho1-2* and *rho1-5* strains to calcofluor white, which binds to the yeast cell wall and is well established to inhibit growth of CWI pathway mutants [58]. Calcofluor white is substantially more inhibitory to *rho1-2* than to *rho1-5* (Figure S8), demonstrating that *rho1-2* is more defective in the CWI pathway than is *rho1-5*. However, C16:1 inhibits *rho1-5* more effectively than it does *rho1-2* (Figure 4F, images g, h, and l). It therefore appears that *rho1-5* causes a greater defect in membrane fluidity signaling, relative to CWI signalling, than does *rho1-2*.

The CWI and membrane fluidity homeostasis pathways presumably possess distinct peripheral components to receive distinct input signals (and to influence distinct effectors, as discussed above). Yeast cell wall damage is sensed by multiple cell wall/PM-embedded sensors (Slg1, Wsc2, Wsc3, Mid2 and perhaps Mtl1) which activate Rom2 (and probably also Rom1) to initiate the CWI pathway (reviewed in [13]). While substantial data link these cell wall sensors to Rom1 and Rom2, no physical or genetic interactions link them to Tus1. Sensors of fluidity, likely to be upstream of either Tus1 or Sac7, have yet to be identified. Tus1, which localizes to the bud neck [57], [59], also participates in the formation of the contractile ring prior to cytokinesis [60]. Moreover, the phosphorylation of Tus1 by a G1 cyclin/CDK complex is important for Rho1 activation at the G1/S boundary and to enable proper actin cytoskeleton polarization [59]. These additional functions of Tus1 suggest that instead of Tus1, perhaps Sac7 transmits a signal to Rho1 which reflects the fluidity status of the PM.

The CWI pathway, and the membrane fluidity homeostasis pathway described here, share at least six components: Rho1, Pkc1, Bck1, Mkk1,2, Mpk1, and Swi6. The overlap between them is comparable to the relationship between three other yeast MAPK pathways: the mating response, the pseudohyphal growth, and the high osmolarity glycerol pathways [11]. The mechanisms by which component-sharing pathways insulate themselves from each others' signals are only beginning to be understood [61]. The sharing of the Rho1/Pkc1/MAPK core components between the CWI and the fluidity homeostasis pathways, by providing a second pair of yeast overlapping MAPK pathways, offers an opportunity to better explore the principles governing insulation.

## Materials and Methods

### Yeast Strains and Growth

Apart from the *rho1* strains shown in Fig. 4F, all strains are derivatives of BY4741 [62]. The deletions borne by the nine yeast strains which were unaffected in growth by C16:1 and C18:1 were each verified by two PCR reactions, each primed by the marker inserted in place of the gene and a region flanking either side of the replaced ORF. The screen and other early experiments used free fatty acids solubilized in 1% tergitol (Sigma-Aldrich). However, subsequent use instead of Na^+^-salts (soaps) of C16:1 and C18:1 eliminated the need for detergent (soaps and free fatty acids were from Nu-Chek Prep, Inc.). To reinforce this distinction, free fatty acid concentrations are expressed as percentages while the concentrations of soaps are expressed as molarity. Benzyl alcohol (>99.8%) and calcofluor white were from Sigma-Aldrich.

### Yeast phospholipid analysis

Yeast from 80 ml of mid-log YPD culture was pelleted (2 min, 1000×g), resuspended in 20 ml water, re-pelleted, and resuspended in water, all at 30°. Three 1 ml aliquots were added to vortexing screw-top glass tubes (Pyrex #9826) containing 4 ml CHCl_3_∶methanol (2∶1); vortexing was continued for 5 min. After centrifugation (as above), discarding the upper phase, addition of 0.8 ml of 0.9% NaCl, vortexing for another minute, and centrifugation once again, the lower phase was withdrawn into a new tube and dried under a stream of N_2_. Each sample was then dissolved in 1 ml CHCl_3_ and applied to a HyperSep SI silica column (Thermo) that had been pre-equilibrated with 3 ml CHCl_3_. The column was then washed with an additional 3 ml CHCl_3_ followed by 5 ml acetone∶methanol (9∶1), discarding the effluent. Phospholipid was then eluted with 3 ml methanol into a third set of tubes. Solvent was removed again under a N_2_ stream and tubes capped overnight. Fatty acid methyl esters were made by resolubilizing in 1 ml methanol∶H_2_SO_4_ (39∶1) and heating at 80° for 2 hr. Water (1.5 ml) and n-hexane (0.1 ml) were added, and after vortexing for 2 min. and centrifugation as above, tubes were chilled in a dry ice/ethanol bath for at least 30 min. A 50 µl aliquot of the liquid hexane phase was then removed into a vial for analysis by gas chromatography/mass spectrometry using the Agilent 5975GC, 6920MS. Fatty acids were identified by NIST Mass Spectral Search Program (version 2.0) and analyzed using MSD ChemStation Data Analysis software.

### Fluorescence Anisotropy

To assess PM fluidity, TMA-DPH fluorescence anisotropy was measured as described previously [47], [63], [64]: 5 ml cultures (grown overnight at 30° in SC complete medium with or without 10 µM sodium palmitoleate or sodium oleate) were diluted to 0.2 OD_600 nm_ and grown in 5 ml SC for 1 hr at 30° and then at room temperature for an additional hour. Cells were pelleted, washed twice with 5 ml 10 mM Tris-HCl, pH 7.0; 1 mM EDTA (TE), and incubated with 0.5 µM TMA-DPH (from Molecular Probes) in TE for 10 min at room temperature. Cells were pelleted again, washed twice with 5 ml TE, resuspended in TE to 0.25 OD_600 nm_ and placed on ice. Immediately prior to analysis, samples were warmed to room temperature and fluorescence anisotropy was measured using an LS50B Perkin Elmer Luminescence Spectrophotometer using FL Winlab software (Perkin Elmer). Excitation and emission wavelengths of 358±5 nm and 430±5 nm were used, respectively, and 10 to 20 anisotropy readings (intensities in the vertical, I_VV_, and horizontal, I_VH_, planes) were obtained every 0.26 sec with G set to 1.58. Anisotropy (*r*_s_) = (I_VV_−GI_VH_)/(I_VV_+2GI_VH_). Values reported were normalized to the *r*_s_ obtained under control conditions (wild type cells grown in the absence of fatty acids; *r*_s_ = 0.306±0.005, *n* = 7).

## Supporting Information

Figure S1 **Example of data from the screen.** Yeast from plate #114 of the MATα deletion collection (one of 52 plates) was applied using a robot to plates containing semi-synthetic medium lacking glucose and containing 1% tergitol, and grown for two weeks at 25°. Plate **A** lacked fatty acids, plate **B** contained 0.1% C18:1 and plate **C** contained 0.1% C16:1. The genes deleted in strains whose growth was influenced by C18:1 and/or C16:1 are shown. For example, growth of *slt2Δ* (top row, 8^th^ column) was C16:1-sensitive yet unaffected by C18:1.
(PDF)

Figure S2 ***pkc1Δ*** **strains readily acquire suppressors of slow growth.** Asci from a sporulated *PKC1/pkc1Δ* strain were dissected on a single plate of YPD medium containing 1 M sorbitol and photographed first at 4 days and then at 14 days of growth at 30°.
(PDF)

Figure S3 **All four MAP3Ks influence C16:1-sensitivity.** *SSK2* and/or *SSK22* were deleted from the *bck1Δ ste11Δ* strain using standard methods to give the two triple and the quadruple deletion strains. Growth was at 30° for 3 days.
(PDF)

Figure S4 **Benzyl Alcohol (BA) and C18:1 counteract the effects of each other on growth.** (A) Growth inhibition of a *pkc1Δ* strain caused by BA is relieved by C18:1. Cells (3, 10-fold serial dilutions) were applied to plates of YPD medium containing 1 M sorbitol and 1% tergitol in the presence (bottom) or absence (top) of BA and in the presence (right) or absence (left) of C18:1 (OA) and grown for 2 days at 30°. (B) Inhibition of growth of a *sap190Δ* strain by C18:1 is suppressed by BA. Cells plated as above on YPD medium containing the indicated levels of C18:1 (NaOA) and/or BA were grown at 30° for 3 days.
(PDF)

Figure S5 **Complete set of growth conditions for** ***rho1*** **strains, some of which are presented in** **Figure 4E** **.**
(PDF)

Figure S6 **All four ratios of acyl group content.** Total PL from each of four strains was purified in multiple experiments and acyl chain content quantified (Table S2 contains the complete data set). Error bars are the standard error of the mean.
(PDF)

Figure S7 **Temperature sensitivity due to** ***ste11Δ*** **is osmo-remedial.** The four plates were incubated at the indicated temperatures for 11 days.
(PDF)

Figure S8 ***rho1-2*** **causes greater calcofluor white-sensitivity than does** ***rho1-5*** **.** The two MAT**a** strains were grown for 3 days on 8 plates at the two temperatures indicated.
(PDF)

Table S1 **Gene deletions which cause oleic acid (OA, C18:1) and/or palmitoleic acid (PO, C16:1) to influence growth.** This data is provided as an Excel file.
(XLS)

Table S2 **PL Acyl Content (Primary Data).**
(PDF)
