# Supplementary figures and images for: Rho Signaling Participates in Membrane Fluidity Homeostasis
Source: PLoS One. 2012 Oct 5;7(10):e45049. doi: 10.1371/journal.pone.0045049 (PMC3465289; doi:10.1371/journal.pone.0045049)

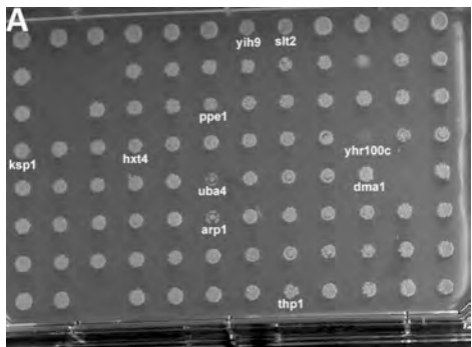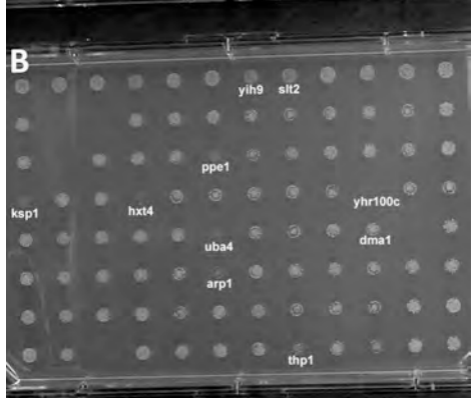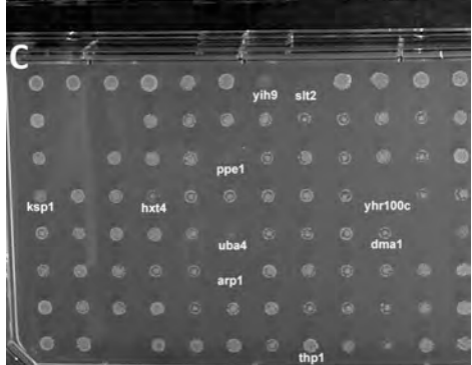

Supplement: Figure S1 — Example of data from the screen. Yeast from plate #114 of the MATα deletion collection (one of 52 plates) was applied using a robot to plates containing semi-synthetic medium lacking glucose and containing 1% tergitol, and grown for two weeks at 25°. Plate A lacked fatty acids, plate B contained 0.1% C18:1 and plate C contained 0.1% C16:1. The genes deleted in strains whose growth was influenced by C18:1 and/or C16:1 are shown. For example, growth of slt2Δ (top row, 8th column) was C16:1-sensitive yet unaffected by C18:1. (PDF) [file pone.0045049.s001.pdf]

**4 days**

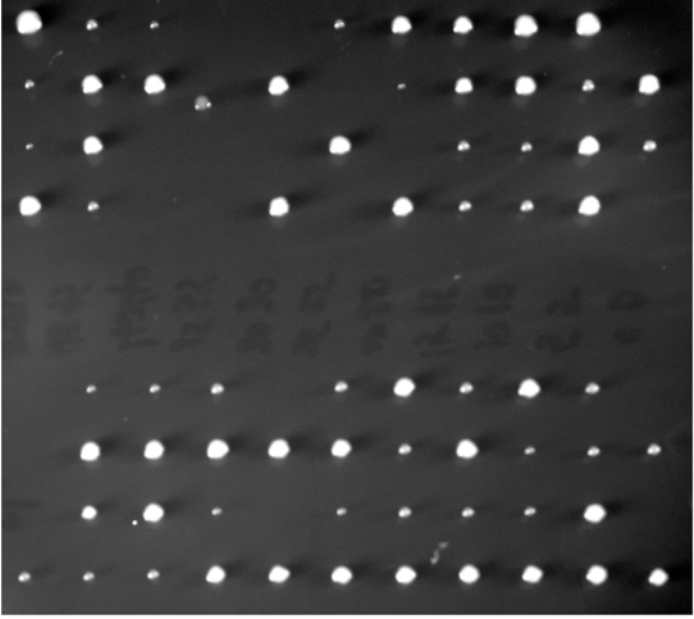

**14 days**

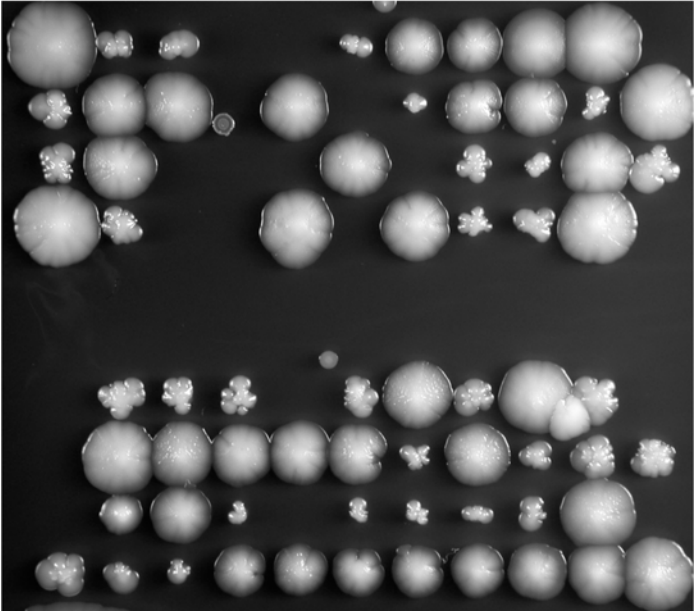

Supplement: Figure S2 — pkc1Δ strains readily acquire suppressors of slow growth. Asci from a sporulated PKC1/pkc1Δ strain were dissected on a single plate of YPD medium containing 1 M sorbitol and photographed first at 4 days and then at 14 days of growth at 30°. (PDF) [file pone.0045049.s002.pdf]

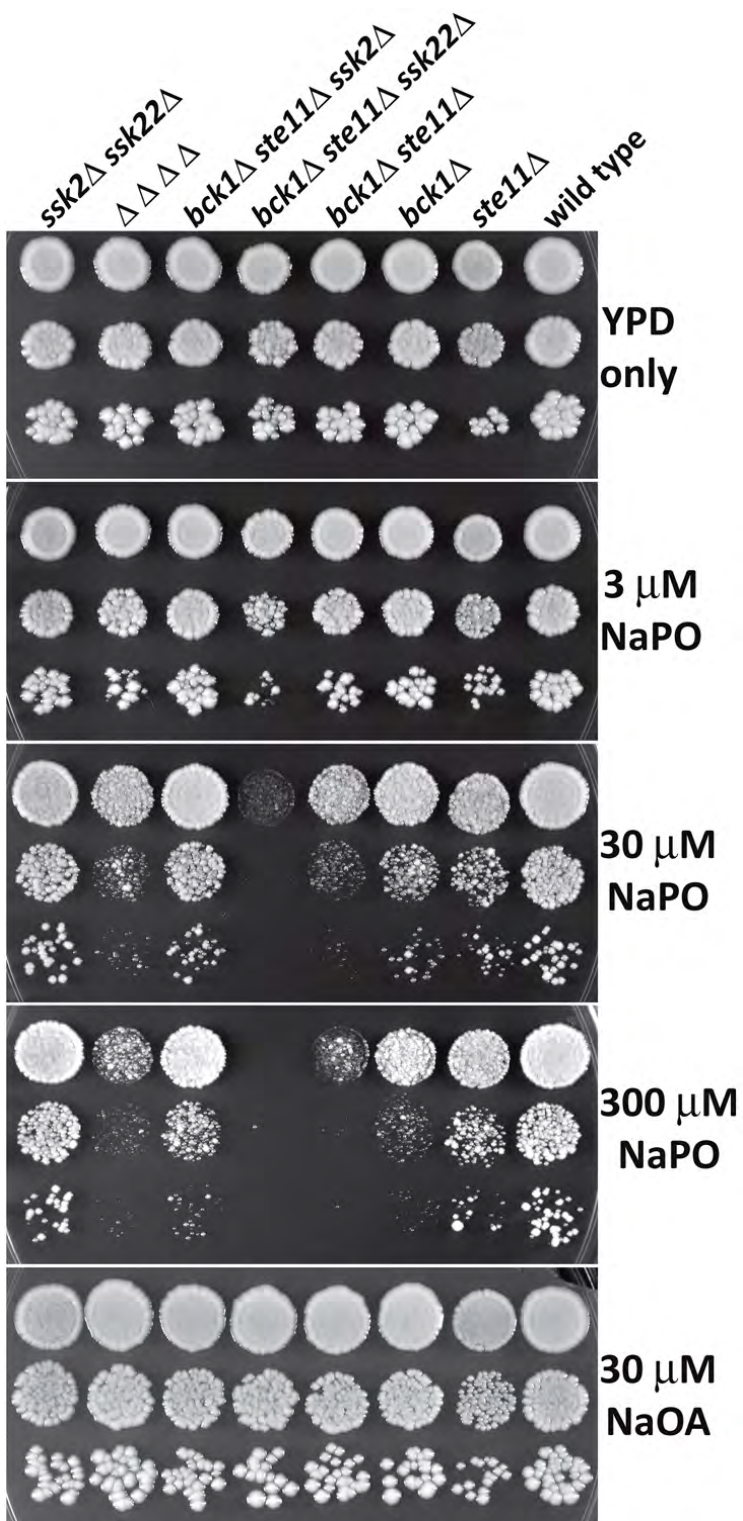

Supplement: Figure S3 — All four MAP3Ks influence C16:1-sensitivity. SSK2 and/or SSK22 were deleted from the bck1Δ ste11Δ strain using standard methods to give the two triple and the quadruple deletion strains. Growth was at 30° for 3 days. (PDF) [file pone.0045049.s003.pdf]

**A**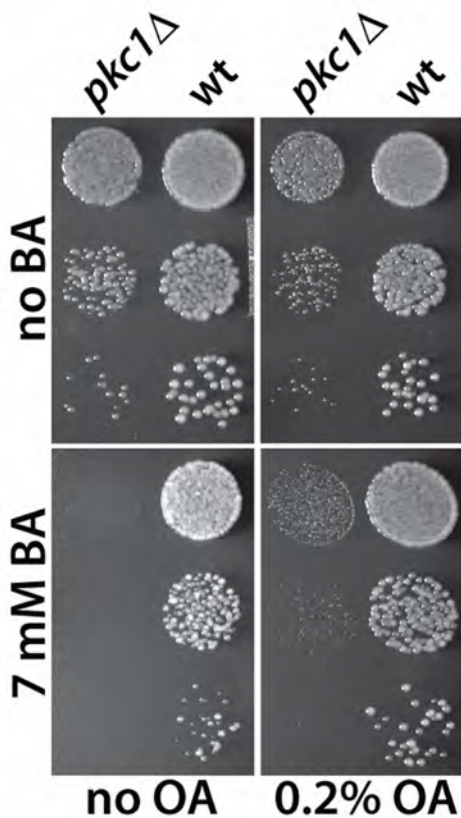**B**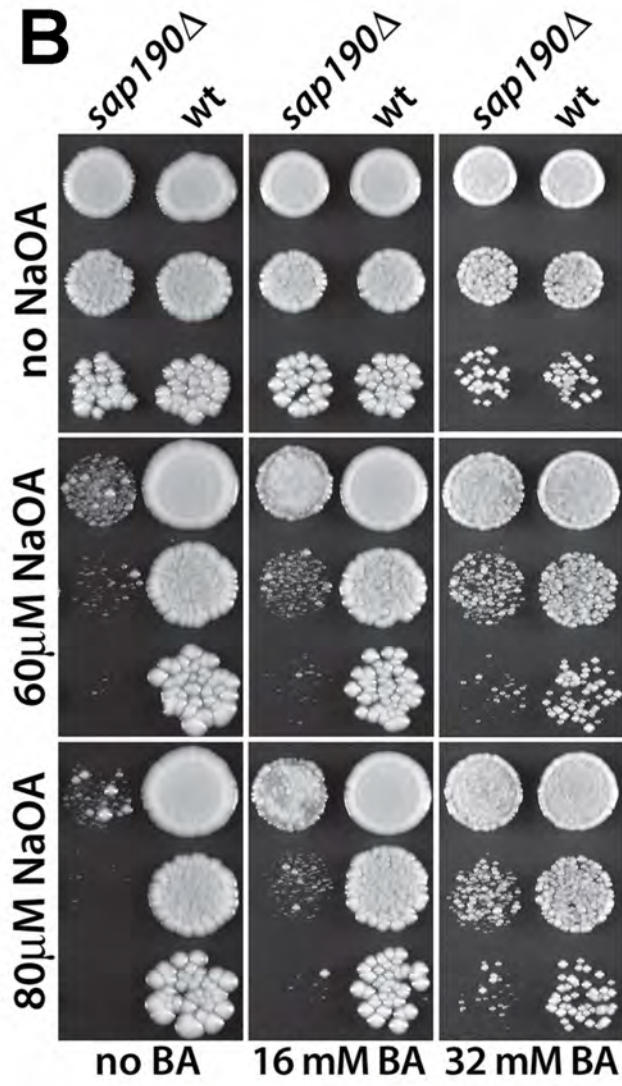

Supplement: Figure S4 — Benzyl Alcohol (BA) and C18:1 counteract the effects of each other on growth. (A) Growth inhibition of a pkc1Δ strain caused by BA is relieved by C18:1. Cells (3, 10-fold serial dilutions) were applied to plates of YPD medium containing 1 M sorbitol and 1% tergitol in the presence (bottom) or absence (top) of BA and in the presence (right) or absence (left) of C18:1 (OA) and grown for 2 days at 30°. (B) Inhibition of growth of a sap190Δ strain by C18:1 is suppressed by BA. Cells plated as above on YPD medium containing the indicated levels of C18:1 (NaOA) and/or BA were grown at 30° for 3 days. (PDF) [file pone.0045049.s004.pdf]

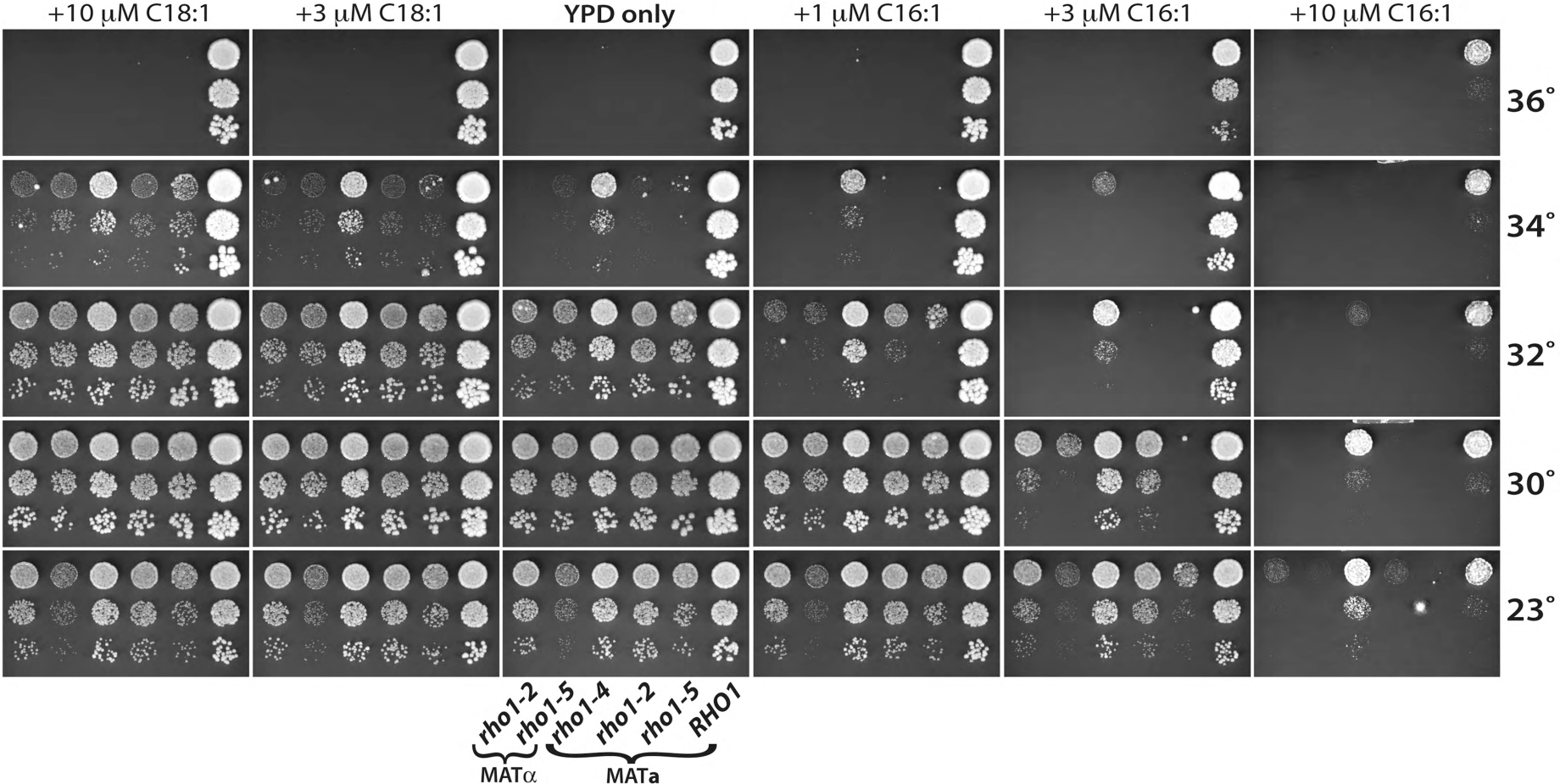

Supplement: Figure S5 — Complete set of growth conditions for rho1 strains, some of which are presented in Figure 4E . (PDF) [file pone.0045049.s005.pdf]

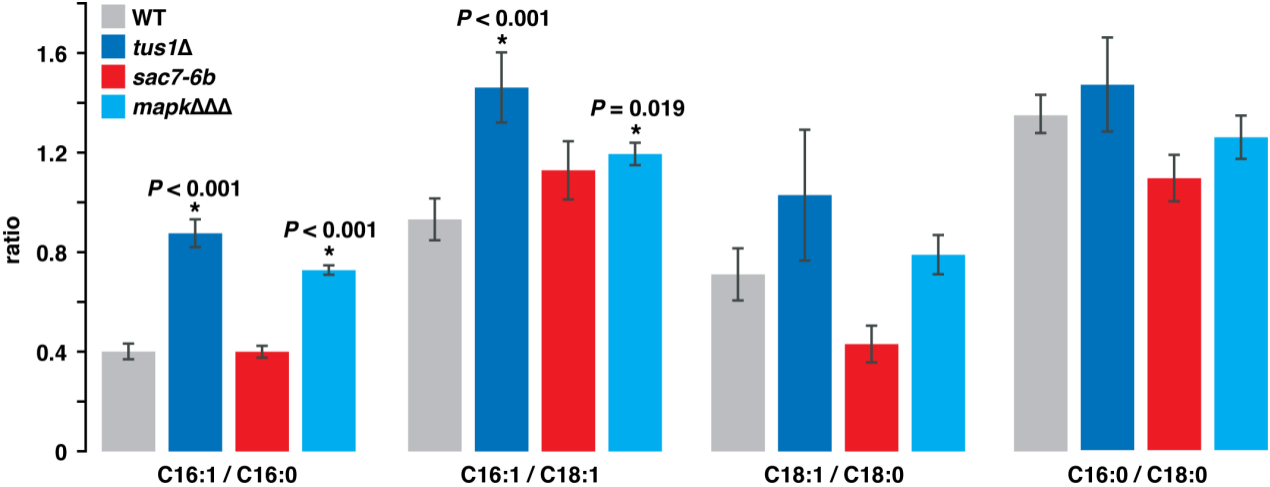

Supplement: Figure S6 — All four ratios of acyl group content. Total PL from each of four strains was purified in multiple experiments and acyl chain content quantified (Table S2 contains the complete data set). Error bars are the standard error of the mean. (PDF) [file pone.0045049.s006.pdf]

*ste11* $\Delta$     *bck1* $\Delta$     wild type    *ste11* $\Delta$     *bck1* $\Delta$     wild type

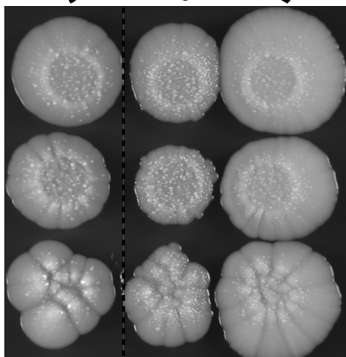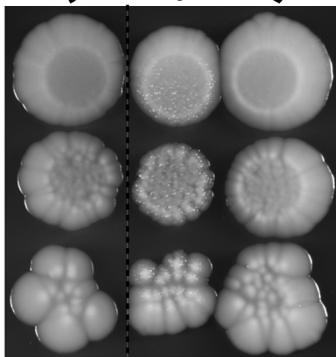

37°

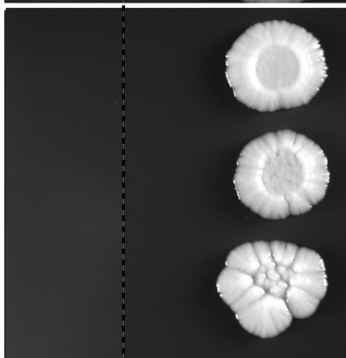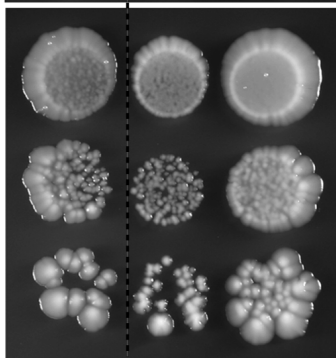

39°

YPD

YPD + 1M sorb.

Supplement: Figure S7 — Temperature sensitivity due to ste11Δ is osmo-remedial. The four plates were incubated at the indicated temperatures for 11 days. (PDF) [file pone.0045049.s007.pdf]

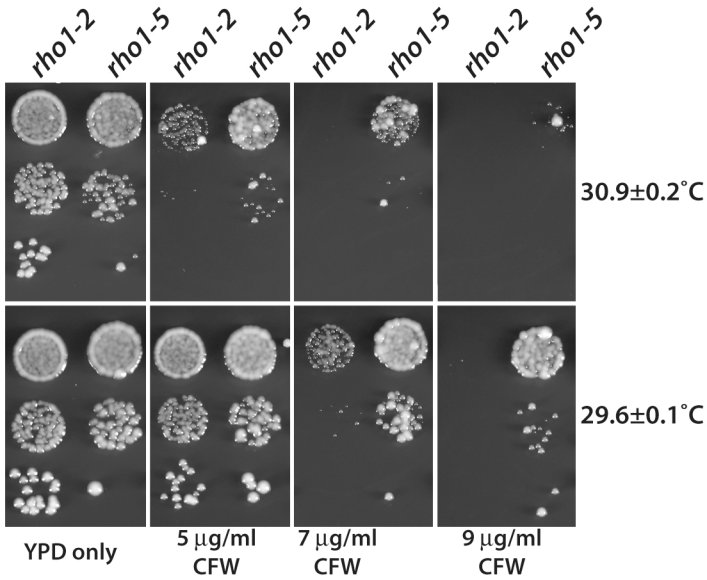

Supplement: Figure S8 — rho1-2 causes greater calcofluor white-sensitivity than does rho1-5 . The two MATa strains were grown for 3 days on 8 plates at the two temperatures indicated. (PDF) [file pone.0045049.s008.pdf]
