# Supplementary material for: Rho Signaling Participates in Membrane Fluidity Homeostasis
Source: PLoS One. 2012 Oct 5;7(10):e45049. doi: 10.1371/journal.pone.0045049 (PMC3465289; doi:10.1371/journal.pone.0045049)
Supplement: Table S2 — PL Acyl Content (Primary Data). (PDF) [file pone.0045049.s010.pdf]

strain	sample	percentages				ratios of percentages				
		C18:0	C18:1	C16:0	C16:1	C16:1/C16:0	C16:1/C18:1	C18:1/C18:0	C16:0/C18:0	C16/C18
wild type	102009-5	41.2	9.8	35.8	13.2	0.37	1.35	0.24	0.87	0.96
	102009-6	29.3	14.9	38.0	17.8	0.47	1.19	0.51	1.29	1.26
	102009-7	21.7	20.3	36.5	21.5	0.59	1.06	0.93	1.68	1.38
	110609-4	29.6	16.6	40.4	13.4	0.33	0.81	0.56	1.37	1.17
	110609-5	34.9	13.8	37.9	13.4	0.35	0.97	0.40	1.09	1.05
	110609-6	30.1	15.9	39.5	14.5	0.37	0.91	0.53	1.31	1.17
	110609-7	24.1	20.6	36.3	19.0	0.52	0.92	0.86	1.51	1.24
	110609-8	41.9	12.4	35.3	10.4	0.29	0.84	0.30	0.84	0.84
	110609-9	45.6	9.2	36.5	8.7	0.24	0.94	0.20	0.80	0.82
	120409-1	19.9	37.8	36.4	6.0	0.16	0.16	1.90	1.83	0.73
	120409-2	22.0	30.5	37.4	10.1	0.27	0.33	1.39	1.70	0.90
	120409-3	23.6	30.2	38.8	7.4	0.19	0.25	1.28	1.64	0.86
	#10 012610	29.0	14.5	36.1	16.6	0.46	1.14	0.50	1.25	1.21
	#11 012610	26.8	15.0	35.7	18.2	0.51	1.22	0.56	1.33	1.29
	#12 012610	30.1	12.6	36.3	17.0	0.47	1.34	0.42	1.21	1.25
	#1 021610	26.5	16.2	34.8	17.7	0.51	1.09	0.61	1.31	1.23
	#2 021610	18.2	17.2	35.0	21.8	0.62	1.27	0.94	1.92	1.61
	#3 021610	25.0	16.4	33.7	16.3	0.48	0.99	0.66	1.35	1.21
	**WT ave**	**28.9**	**18.0**	**36.7**	**14.6**	**0.40**	**0.93**	**0.71**	**1.35**	**1.12**
	WT s.e.m.	1.8	1.8	0.4	1.1	0.03	0.08	0.10	0.08	0.05
tus1	120409-7	13.0	29.9	28.3	28.8	1.02	0.97	2.30	2.18	1.33
	120409-8	14.9	24.5	31.0	29.6	0.95	1.21	1.64	2.08	1.54
	120409-9	13.6	28.5	29.1	28.8	0.99	1.01	2.09	2.14	1.38
	#1 012610	21.9	15.2	29.2	29.5	1.01	1.94	0.70	1.34	1.58
	#2 012610	31.7	12.9	29.6	22.4	0.76	1.74	0.41	0.93	1.17
	#3 012610	18.4	18.2	29.3	29.5	1.01	1.62	0.99	1.59	1.61
	#10 021610	41.5	9.4	30.1	16.4	0.55	1.75	0.23	0.72	0.91
	#11 021610	35.8	10.6	31.0	22.1	0.71	2.08	0.30	0.87	1.14
	#12 021610	23.7	14.5	32.3	28.8	0.89	1.98	0.61	1.36	1.60
	**tus1 ave**	**23.8**	**18.2**	**30.0**	**26.2**	**0.88**	**1.46**	**1.03**	**1.47**	**1.36**
	tus1 s.e.m.	3.3	2.4	0.4	1.5	0.06	0.14	0.26	0.19	0.08
sac7	#7 012610	25.4	14.5	37.7	17.6	0.47	1.21	0.57	1.49	1.39
	#8 012610	36.5	11.7	35.1	11.8	0.34	1.01	0.32	0.96	0.97
	#9 012610	30.6	14.2	35.1	16.5	0.47	1.17	0.46	1.15	1.15
	#4 021610	39.5	8.2	35.0	12.3	0.35	1.51	0.21	0.89	0.99
	#5 021610	26.6	18.5	31.2	11.8	0.38	0.64	0.70	1.17	0.95
	#6 021610	26.4	8.7	23.8	9.5	0.40	1.09	0.33	0.90	0.95
	**sac7 ave**	**30.8**	**12.6**	**33.0**	**13.2**	**0.40**	**1.13**	**0.43**	**1.09**	**1.07**
	sac7 s.e.m.	2.4	1.6	2.0	1.3	0.02	0.12	0.07	0.09	0.07
bck1 ste11 ssk22	102009-1	29.4	17.7	29.7	23.1	0.78	1.30	0.60	1.01	1.12
	102009-2	28.4	17.6	30.5	23.4	0.77	1.33	0.62	1.08	1.17
	102009-3	28.4	17.6	30.5	23.4	0.77	1.33	0.62	1.08	1.17
	110609-1	15.8	25.4	32.6	26.3	0.81	1.03	1.61	2.06	1.43
	110609-2	28.5	24.5	28.7	18.3	0.64	0.75	0.86	1.01	0.89
	110609-3	24.4	20.4	32.1	23.2	0.72	1.14	0.84	1.32	1.24
	110609-10	29.9	18.3	31.6	20.2	0.64	1.11	0.61	1.06	1.08
	#4 012610	22.5	18.5	31.3	23.7	0.76	1.28	0.82	1.39	1.34
	#5 012610	24.8	17.9	31.1	22.6	0.73	1.26	0.72	1.26	1.26
	#6 012610	33.4	14.1	31.0	18.0	0.58	1.27	0.42	0.93	1.03
	#7 021610	25.3	18.3	29.5	21.6	0.73	1.18	0.72	1.17	1.17
	#8 021610	22.3	19.5	30.0	23.8	0.79	1.22	0.87	1.35	1.29
	#9 021610	19.6	18.5	32.3	24.4	0.75	1.32	0.94	1.65	1.49
	**3D ave**	**25.6**	**19.1**	**30.8**	**22.5**	**0.73**	**1.19**	**0.79**	**1.26**	**1.21**
	3D s.e.m.	1.3	0.8	0.3	0.7	0.02	0.05	0.08	0.09	0.05
